# Feasible Production of Lignans and Neolignans in Root-Derived In Vitro Cultures of Flax (*Linum usitatissimum* L.)

**DOI:** 10.3390/plants9040409

**Published:** 2020-03-25

**Authors:** Sumaira Anjum, Amna Komal, Samantha Drouet, Humera Kausar, Christophe Hano, Bilal Haider Abbasi

**Affiliations:** 1 Department of Biotechnology, Kinnaird College for Women, Lahore-54000, Pakistan; aaykay28@gmail.com (A.K.); humera.kausar@kinnaird.edu.pk (H.K.); 2 Laboratoire de Biologie des Ligneux et des Grandes Cultures, INRA USC1328/Université d’Orléans, 28000 Chartres, France; samantha.drouet@univ-orleans.fr; 3 Department of Biotechnology, Quaid-i-Azam University, Islamabad-45320, Pakistan

**Keywords:** flax, neolignans, lignans, adventitious root, callus culture, antioxidant activity

## Abstract

Flax lignans and neolignans impart health benefits, particularly in treating different types of cancers, due to their strong phytoestrogenic and antioxidant properties. The present study enhances the comprehension on the biosynthesis of antioxidant lignans and neolignans in root-derived in vitro cultures of flax (both callus and adventitious root). The results presented here clearly showed that the adventitious root culture efficiently produced a higher amount of lignans (at day 40) and neolignans (at day 30) than callus culture of flax. High performance liquid chromatography (HPLC) analysis revealed that the accumulations of secoisolariciresinol diglucoside (SDG, 5.5 mg g^−1^ DW (dry weight)) and dehydrodiconiferyl alcohol glucoside (DCG, 21.6 mg/g DW) were 2-fold higher, while guaiacylglycerol-β-coniferyl alcohol ether glucoside (GGCG, 4.9 mg/g DW) and lariciresinol glucoside (LDG, 11.9 mg/g DW) contents were 1.5-fold higher in adventitious root culture than in callus culture. Furthermore, the highest level of total phenolic production (119.01 mg/L), with an antioxidant free radical scavenging activity of 91.01%, was found in adventitious root culture at day 40, while the maximum level of total flavonoid production (45.51 mg/L) was observed in callus culture at day 30 of growth dynamics. These results suggest that adventitious root culture can be a good candidate for scaling up to industrial level to commercially produce these pharmacologically and nutritionally valuable metabolites.

## 1. Introduction

**Figure 1:**
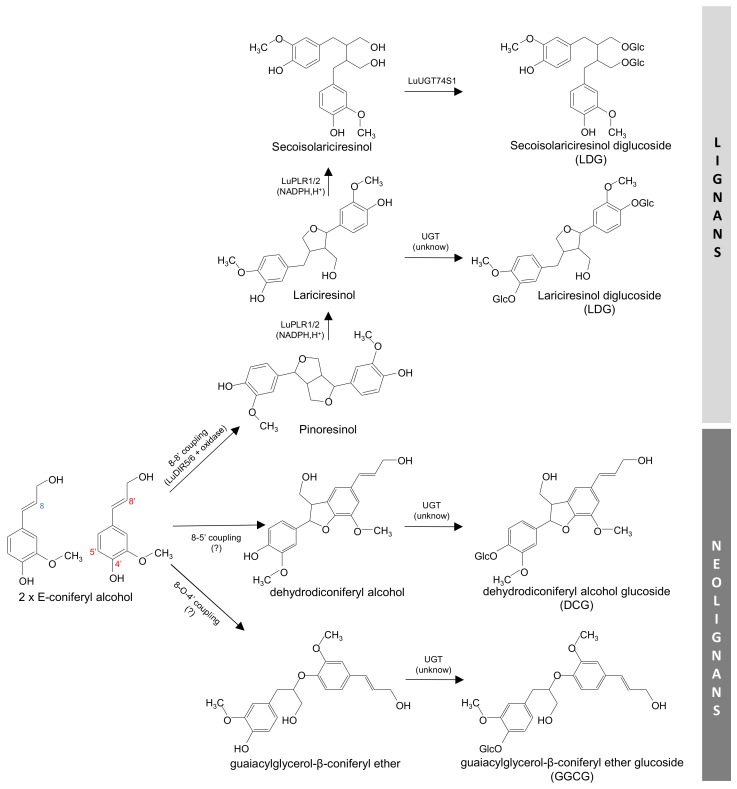
Biosynthetic pathway of major lignans and neolignans detected in flax root-derived tissues by RP-HPLC (Reverse Phase High Performance Liquid Chromatography). Abbreviations: dirigent protein (DIR); pinoresinol-lariciresinol reductase (PLR); UDP-glucosyltransferase (UGT).

Flax (*Linum usitatissimum* L.) is one of the oldest annual crops and presents numerous benefits to human health [1,2,3,4,5]. Its average production of 1.6 million metric tons of flax produced worldwide shows that its economic importance is still relevant [6]. Flaxseed is a valuable source that contributes to a nutritionally rich diet, as it contains various dietary components to be considered as a functional food [7]. Among the nutritionally important components of flaxseed omega-3 fatty acids, polyphenols (in particular, lignans), proteins and dietary fibers are included [7]. Flax has also been served for medicinal purposes for a long time, as it contains medicinally important lignans, which demonstrate pronounced antioxidant and phytoestrogenic activities [1,2,3]. The pharmacologically active lignans in flaxseeds include secoisolariciresinol (SECO), lariciresinol diglucoside (LDG) and secoisolariciresinol diglucoside (SDG) (Figure 1), which represent over 95% of the total lignan components from flaxseeds [8,9]. Following their ingestion, the intestinal bacteria metabolizes flax lignans into the mammalian lignans (enterolactone and enterodiol), reported to possibly reduce the incidence of different cancers, especially the colon, breast and prostate cancers [3]. In addition to the lignans accumulated in its seeds, flax in vitro cultures have been reported as a rich source of antioxidant neolignans (dehydrodiconiferyl alcohol glucoside, DCG and guaiacylglycerol-β-coniferyl alcohol ether glucoside, GGCG) (Figure 1) [10,11,12,13,14,15,16].

Lignans and neolignans share a common biosynthesis pathway, which involves the dimerization of two units of E-coniferyl alcohol, as shown in Figure 1. The early biosynthetic steps of lignans’ formation have been well characterized [17]. First, a dirigent protein (DIR) (LuDIR1, LuDIR5 or LuDIR6 for in flax) directs the stereoselective radical-radical coupling of two E-coniferyl alcohol radical units into pinoresinol [18,19,20]. Pinoresinol is then reduced into secoisolariciresinol by a stereoselective pinoresinol-lariciresinol reductase (PLR) enzyme, via lariciresinol formation. Two PLRs with opposite stereospecificity have been cloned and characterized in flax [20,21,22,23,24,25,26,27]. In flax, lariciresinol and secoisolariciresinol are stored as glycosides obtained after the action of specific UDP-glucosyltransferases [28]. Less is known about neolignans biosynthesis. Flax have been reported to actively produce several neolignans, in particular DCG and GGCG [10,12,13,14,15,16], and their accumulations have been related to the expression of some DIR genes in flax hairy root cultures [19].

Since lignans and neolignans have significant physiological, pharmacological and nutritional properties, further studies are required to be carried out to develop a better understand about their biosynthesis, regulation and accumulation in different plant species and culture systems [29]. Although a significant accumulation of lignans has been reported in flax roots [19,30] and root-derived in vitro cultures (mainly hairy roots) [19,31,32,33], their production in roots has been much less studied than in seeds. Moreover, having a shallow root system, flax produce less root biomass than other crops [34]. Plant in vitro tissue culture platform is an alternative for producing pharmacologically and nutritionally important metabolites, when their natural supply or extraction from natural resources is limited [35,36], and can be an attractive alternative for the production of lignans and neolignans from roots in flax. Adventitious root culture is a better choice for the sustainable and stable production of commercially important plant metabolites, as compared to other in vitro cultures of plants [37,38,39,40]. Adventitious roots have high proliferation rates and are easier to maintain than other cultures [41,42]. Moreover, contrary to hairy root culture, adventitious root cultures are genetically stable and do not produce any toxic chemicals (opines) [43]. Flax is mostly utilized for improved the production of linseed oil and fiber; on the contrary, it is less exploited for the greater production of antioxidant lignans and neolignans. Therefore, the establishment of various in vitro cultures of flax to produce these health-promoting secondary metabolites should be a focus of intense research. To date, the adventitious root formation of flax for lignans and neolignans has never been evaluated.

In this research study, the main objective was to establish in vitro cultures of flax derived from roots which are efficient in producing higher levels of antioxidant polyphenols, neolignans and lignans. The in vitro adventitious root cultures of flax were established and their growth dynamics and secondary metabolite production were compared to root-derived callus cultures. The main lignans (SDG and LDG) and neolignans (DCG and GGCG) accumulated in flax in vitro cultures were quantified by using RP-HPLC (Reverse Phase High Performance Liquid Chromatography). Furthermore, the productions of total phenolic and total flavonoid, as well as the antioxidant potential of flax, were also evaluated during the different growth phases of callus and adventitious root cultures. As per our knowledge, this is the first comprehensive report on the feasible production of GGCG, SDG, DCG and LDG in adventitious root culture of flax which can be potentially up-scaled to enhance the production of these valuable secondary metabolites on an industrial level.

## 2. Results and Discussion

### 2.1. Establishment and Growth Dynamics of Callus Culture

The roots of *L. usitatissimum* are a rich source of secondary metabolites which have potential anti-cancerous properties [3,4]. Therefore, in the preliminary experiments, callus culture was established by inoculating the root explant on MS media supplied with different concentrations (0.5–3.0 mg/L) of NAA, 2,4-D, IBA and IAA. The optimum concentration of PGRs which produced a high quantity of precious metabolites in callus culture of *L. usitatissimum* was determined by the different growth parameters of calli (Table 1). The treatment of different PGRs affected the minimum number of days required for callus initiation and percentage callogenesis. The highest percentage callogenesis (98%) with the minimum number of days required (7 days) for callus initiation was found in the case of 1.0 mg/L treatment of NAA. The cultures treated with IBA resulted in the lowest callogenic frequencies. These results were in coherence with Janowicz et al. [44] observing a higher frequency of callogenesis in a media supplied with low concentrations of NAA (0.1–2.0 mg/L). There was no callus formation on PGR-free medium (MS0; control). Calli produced in response to various treatments of PGRs were harvested after five weeks of culture, and the highest value of callus production as fresh weight (FW) (392.76 g/L) and dry weight (DW) (13.26 g/L) was also associated with the MS medium supplemented with 1.0 mg/L NAA (Table 1). It can be concluded that NAA (1.0 g/L) is suitable for inducing callus as compared to other growth hormones, and this is also supported by other studies on callogenesis frequency and biomass production in *L. usitatissimum* [35,44]. In agreement with our data, there are numerous reports on other medicinally important plant species (e.g., [45]). Data of various growth parameters showed that treatment with a 1.0 mg/L concentration of NAA was the best for the highest rate of callogenesis and the accumulation of the maximum biomass callus culture of *L. usitatissimum*, therefore, callus induced in response to the above mentioned concentration of NAA was selected to investigate the synthesis of secondary metabolites during different phases of growth.

**Figure 2:**
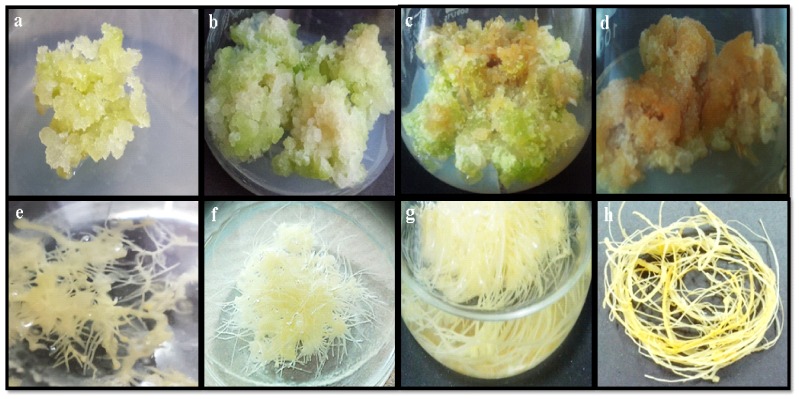
Different growth phases of callus (1.0 mg/L NAA) and adventitious root (0.5 mg/L NAA) cultures of flax. (**a**) Lag phase, (**b**) exponential phase, (**c**) stationary phase and (**d**) decline phase of callus culture, (**e**) log phase, (**f**) exponential phase and (**g**) stationary phase of adventitious root culture, and (**h**) harvested roots per flask.

**Figure 3:**
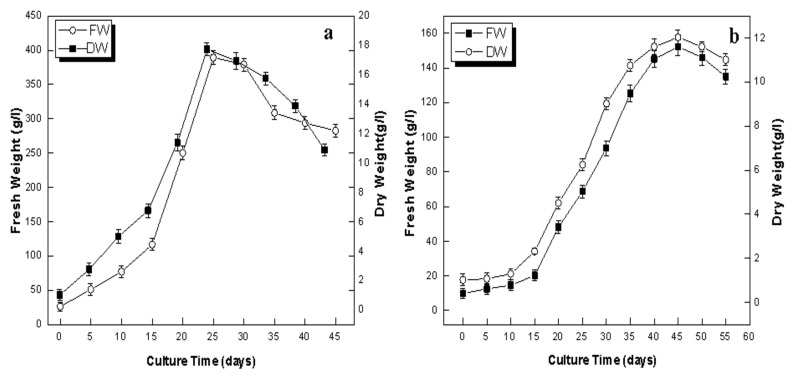
Growth dynamics of biomass accumulation in in vitro cultures of flax. (**a**) Callus culture (**b**) adventitious root culture. Values are mean ± SE of three replicates.

The callus growth dynamics allow the identification of the appropriate moment for callus booming and observing the accumulation of phase-dependent primary and secondary metabolites [46]. In this research, growth dynamics and the biomass accumulation of callus were studied for 45 days with an interval of five days. The different growth phases of callus (1.0 mg/L NAA) are shown in Figure 2a–d. The lag, exponential and stationary growth phases were characterized, empowering the calculation of the doubling time of biomass production. When initializing with the inoculum (26.25 g/L), doubling in FW with the values of 77.01 g/L was witnessed on day 10, while highest fresh biomass accumulation with the values 389.0 g/L (14-fold) was reported on day 25 of culture (Figure 3a). Similar trends in biomass accumulation in the callus (0.5 BA & 1.0 mg/L NAA) of *L. usitatissimum* were also found by Siegen et al. [35] The growth dynamics showed that both the FW and DW increase up to day 25 (log phase), but later on there was a gradual decrease in biomass from day 25 to 45 (stationary and death phase). These results showed that NAA significantly enhanced biomass accumulation during the log phase of callus growth dynamics. These observations have similarities with the results of Raj et al. [46] in the callus culture of *Securinega suffruticosa*.

### 2.2. Establishment and Growth Dynamics of Adventitious Root Culture

In preliminary experiments, root explants were cultured on MS liquid medium supplied with IAA, NAA and IBA for adventitious roots formation. The PGR which allowed maximum synthesis of valued-added secondary metabolites in adventitious roots of *L. usitatissimum* was determined by different growth parameters of adventitious root culture (Table 2). Different PGRs treatments concerning minimum days required for the induction of adventitious roots, percentage rooting and maximum biomass production, 0.5 mg/L NAA was proved to be the best suited treatment, having 100% rooting frequency, the minimum number of days required for root induction (eight days) and highest yield of biomass (FW; 123.21 g/L, DW; 10.52 g/L) after five weeks of culture. The different growth phases of adventitious root (0.5 mg/L NAA) cultures are shown in Figure 2e–h. It is evident from many previous reports that the induction of the adventitious root is prompted by the addition of NAA to MS medium in various medicinally important plant species [40,42]. In our research, an inverse relationship was observed between biomass accumulation and auxin concentrations. As the concentration of auxins (for any auxin tested) increased from 0.5 to 3.0 mg/L in media, it led to the a decrease in biomass accumulation. Lower levels (0.5 mg/L) of each auxin applied resulted in highest percentage rooting and biomass accumulation, as compared to the high levels of all auxins applied (1.0–3.0 mg/L; Table 2). We can speculate that the high levels of auxins might result in the inhibition of key enzymes required for the initiation of adventitious roots formation. Moreover, the addition of high levels of auxins in media often resulted in low levels of secondary metabolite accumulation [43]. The results of Reis et al. [37] support our results that a lower level (9.7 µM) of NAA speeds up adventitious root formation in *Stevia rebaudiana*. Khan et al. [39] also observed that 1.0mg/L NAA was responsible for maximum biomass production and rooting percentage in the root culture of *Silybum marianum*. NAA has shown superiority, as compared to other auxin, in producing adventitious roots in *Withania somnifera* [42]. This is probably due to the fact that in in vitro cultures, plant cells and tissues have a greater tendency to absorb and utilize NAA rather than IAA and IBA [43].

Our results showed that among all the tested auxins, NAA (0.5 mg/L) was best suited for the induction, growth and development of adventitious root culture. Therefore, the growth dynamics of adventitious root culture were established to find out the exact stage of maximum accumulation of bioactive secondary metabolites (Figure 3b). The growth curve of adventitious root culture was characterized by a lag phase of 10 days, followed by an exponential phase of 35 days (from 15 to 45 days), and a subsequent stationary phase of only five days. Stationary phase lengths were shorter in time, which ultimately declined towards the death phase. Adventitious root culture showed a retarded enhancement in biomass accumulation at the start of the log phase and reached their highest levels of 152.12 g/L FW and 12.02 g/L DW at the 45th day of culture (Figure 3b). Increases of 15-fold in FW and 12-fold in DW were observed when compared with initial biomass of inoculum. Similarly, Gabr et al. [31] reported an eight-fold increase in the DW of the hairy roots of *L. usitatissimum* and Kim et al. [47] observed a 12-fold increase in the DW of *P. gingeng* adventitious root culture, at peaks of their respective exponential growth phases. It is evident from the above results that the adventitious root culture of *L. usitatissimum* can accumulate high levels of biomass and can serve as a promising source of large-scale biomass production.

### 2.3. Phenolics Production in Callus and Adventitious Root Culture

**Figure 4:**
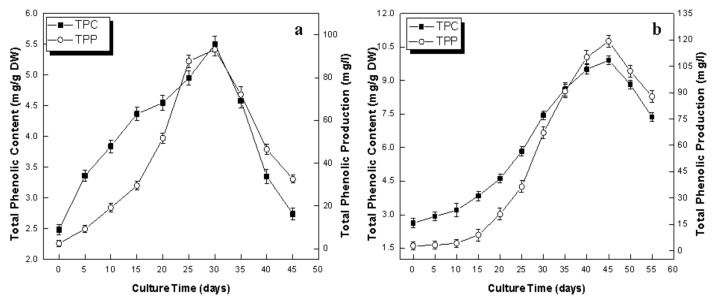
Dynamics of accumulation of total phenolic content (TPC) and total phenolic production (TPP) in in vitro cultures of flax. (**a**) Callus culture (**b**) adventitious root culture. Values are mean ± SE of three replicates.

Phenolic compounds are an important class of naturally occurring antioxidant secondary metabolites. In the present study, the dynamics of phenolic production in callus culture and adventitious root culture of *L. usitatissimum* indicated that the root culture was more efficient in productions of phenolics than callus culture. The profiles of the TPC and TPP in callus and adventitious root culture are shown in Figure 4a and b, respectively. In the case of callus culture, TPC and TPP increased linearly with the culture duration and reached their maximum values of 5.50 mg/g DW and 93.18 mg/L, respectively, on the 30th day of culture (Figure 4a). Meanwhile, in the case of adventitious root culture, maximum values of TPC (9.91 mg/g DW) and TPP (119.01 mg/L) were seen on day 45 of culturing (Figure 4b). Adventitious root culture showed an almost two-fold increase in TPC in comparison with callus culture. Likewise, Gabr et al. [31] also observed that the hairy root culture of *L. usitatissimum* showed a higher amount of phenolic compounds in comparison to callus culture. Recently, the same authors reported that the transformed hairy root culture of *L. usitatissimum* accumulated higher concentration of TPC (40.02 mg/g DW) as compared to non-transformed hairy root culture [32]. Zhao et al. [48] also reported that the cell culture of *Catharanthus roseus* produced a higher amount of phenolic compounds (alkaloids) than its compact callus culture. No report is present on the production of phenolic compounds in the adventitious root culture of *L. usitatissimum* until now. However, recently many researchers reported an improvement in the production of phenolics and flavonoids in elicited-cell cultures of *L. usitatissimum* [13,14,15,16,49], which also endorsed our results. Moreover, many researchers also reported that adventitious root cultures were more potent than callus cultures in the production of phenolic compounds [50]. This might be because of different culture conditions (liquid/solid media), or due to different types of cultures i.e., undifferentiated tissue (callus culture) or differentiated tissue (root culture) [51]. Differentiated tissues have the ability to efficiently transport nutrients and oxygen across the interior of the liquid media. It also affects the secondary metabolism, as some extent of differentiation is necessary to produce and store some types of secondary metabolites [31,52].

### 2.4. Flavonoids Production in Callus and Adventitious Root Culture

**Figure 5:**
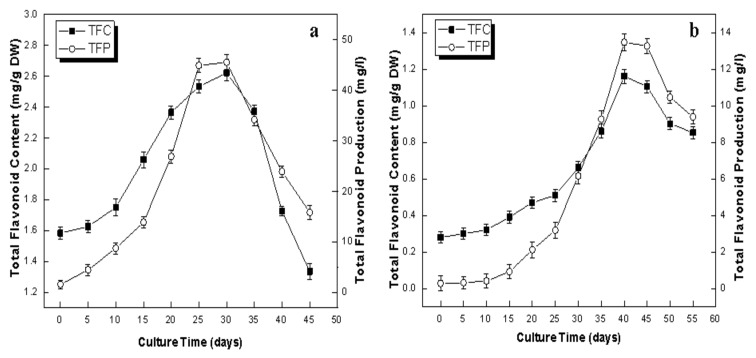
Dynamics of accumulation of total flavonoid content (TFC) and total flavonoid production (TFP) in in vitro cultures of Flax. (**a**) Callus culture, (**b**) adventitious root culture. Values are mean ± SE of three replicates.

A plethora of biological activities have made flavonoids a very attractive class of phytochemicals for the prevention and treatment of many human diseases and also driven researchers to find alternative sources of production other than conventional field cultivation [53]. In the present study, the dynamics of TFC and TFP in the adventitious root and callus culture of *L. usitatissimum* have been established. The profile of the TFC and TFP in callus culture showed their peak values of 2.62 mg/g DW and 45.51 mg/L, respectively, during log phase (30th day; Figure 5a). Meanwhile, in the case of adventitious root culture, the maximum values of TFC (1.16 mg/g DW) and TFP (13.47 mg/L) were witnessed on the 45th day of culture (Figure 5b). Callus culture showed the maximum accumulation of TPC and TFC at the same day (30th day) of culture, while adventitious root showed the maximum accumulation of TPC at 45th day and TFC at 40th day of culture. Contrary to TPP profiles, callus culture showed an almost three-fold increase in TFP as compared to adventitious culture. Similarly, higher flavonoids production was reported in *Hemidesmus indicus* callus culture than in its adventitious root culture [54]. These results are also in agreement with our previous report in which the stem-derived callus showed a high value of TFC (2.72 mg/g DW) as compared to the in vitro-derived whole plantlets (1.61 mg/g DW) of *L. usitatissimum* [55]. Similarly, the highest levels of flavonoids production was reported during the log phase of callus cultures of various medicinal species [54,56]. Production of flavonoids in tissue cultures was reported to be more effective in callus cultures than other organ cultures [52]. In our study, the flavonoids were produced at a low level in adventitious root culture, which could be due to the specific growth stages while adventitious roots were developing. The growth stage influences the activity of l-phenylalanine ammonia-lyase (PAL), an enzyme of key importance in the biosynthesis pathway of the flavonoid. Moreover, the PAL activity is also dependent on the developmental stage, plant tissue type and genotype [39].

### 2.5. Antioxidant Activity of Callus and Adventitious Root Cultures

**Figure 6:**
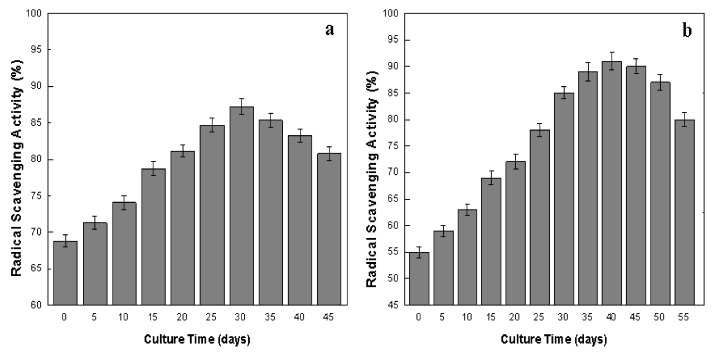
Dynamics of antioxidant activities of in vitro cultures of flax. (**a**) Callus culture, (**b**) adventitious root culture. Values are mean ± SE of three replicates.

DPPH assay is a cost-efficient, simple, sensitive and widely used method for the evaluation of anti-oxidant activity of products derived from plant cells [51,55]. In our research, the highest value of DPPH free radical scavenging activity (FRSA) was 87.23% at day 30 of callus culture (Figure 6a). Meanwhile, in adventitious root culture, the highest FRSA (91.01%) was recorded at day 40 of culture (Figure 6b). In both cultures, FRSA was dependent on secondary metabolites production, but independent of biomass accumulation during different growth phases. In callus culture, the highest antioxidant activity (87.23%) and maximum TPP (93.18 mg/L) and TFP (45.51 mg/L) were recorded on the same day (30th) of culture. Similarly, in adventitious root culture, the highest antioxidant activity (91.01%) and maximum TPP (119.01 mg/L) was at day 40, while the maximum biomass accumulation (DW; 12.02 g/L) was seen at day 45 of culture. These results showed an association between secondary metabolites and antioxidant activity in the callus and adventitious root cultures of *L. usitatissimum*. Adventitious roots showed greater antioxidant activity than callus culture, probably due to higher production of phenolics than callus culture. Studies on the antioxidant properties of in vitro cultures of *L. usitatissimum* are scarce, and only a few reports are available on the antioxidant activity of its callus, hairy root cultures and cell suspension [13,14,15,16,31,32,49]. These investigators reported higher antioxidant activity in hairy root and cell suspension cultures than the callus culture of flax [31]. Szewczyk et al. [4] reported an association between the secondary metabolites (phenolics) and the anticancerous activity of *L. usitatissimum* root extract against breast cancer cell lines. Recent studies show that phenolics enhance the antioxidant activities in the in vitro cultures of many medicinal plants, as compared to wild plants [48,57]. Our study indicates that the major antioxidants could be polyphenols present in the in vitro cultures of *L. usitatissimum* and which can substitute the rare natural resources of food and medicine.

### 2.6. Production of Lignans in Callus and Adventitious Root Cultures

**Figure 7:**
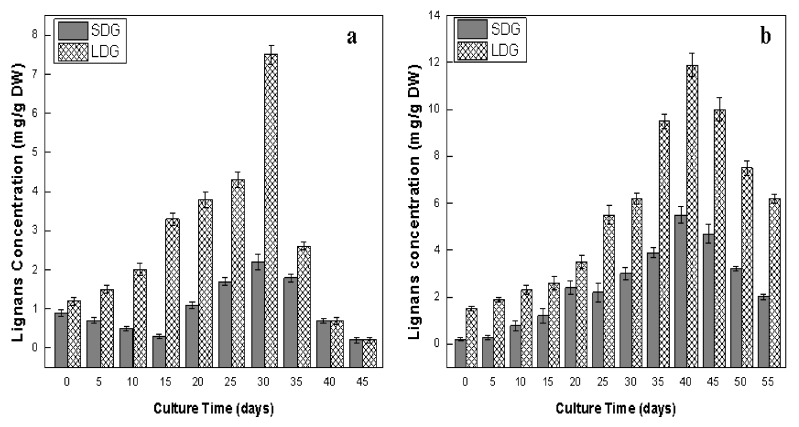
Dynamics of biosynthesis of lignans (secoisolariciresinol diglucoside; SDG and lariciresinol diglucoside; LDG) in in vitro cultures of flax. (**a**) Callus culture, (**b**) adventitious root culture. Values are mean ± SE of three replicates.

**Figure 8:**
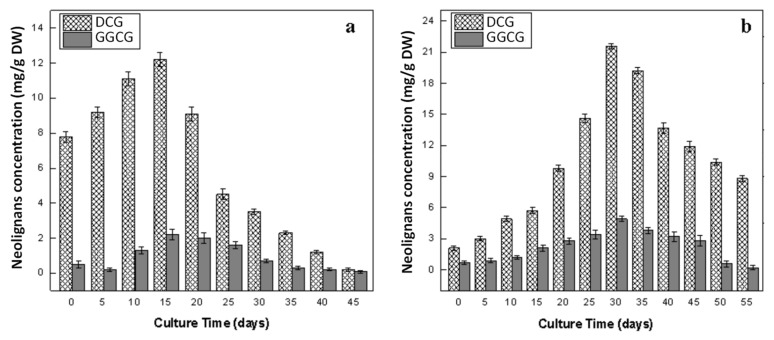
Dynamics of biosynthesis of neolignans (dehydrodiconiferyl alcohol glucoside; DCG and guaiacylglycerol-β-coniferyl alcohol ether glucoside; GGCG) in in vitro cultures of flax. (**a**) Callus culture, (**b**) adventitious root culture. Values are mean ± SE of three replicates.

Quantification of pharmacologically and nutritionally important lignans (SDG and LDG) and neolignans (DCG and GGCG) was carried out by RP-HPLC analysis (Figure 7 and Figure 8). In flaxseeds, lignans are stored in the form of an ester-linked complex coupled with hydroxymethyl glutaric acid [17,58]. On the contrary, in roots, lignans are not presented in the form of a complex, but free and stored as glycosides [19,33,59]. The presence of both lignans and neolignans have been previously reported in the roots of flax plants grown in a greenhouse [30], but also in root-derived in vitro cultures (both callus and hairy roots) [19,31,32,33,59,60]. Both SDG and DCG have been previously reported in root-derived callus [22,60,61,62,63,64] and hairy root cultures [19,31,32,33,59], with SDG content ranging from 1.4 mg/g DW [32] to 13.8 mg/g DW after elicitation treatment [65], and DCG content ranging from 7.83 mg/g DW [19,59] to 47.7 mg/g DW [61]. The SDG and DCG contents observed in our root-derived callus and adventitious root cultures are in the same range. The lignan LDG and the neolignan GGCG have been previously reported in flax callus and cell suspension [11,12,13,14,15,16,66], ranging from 2.2 mg/g DW [67] to 21.6 mg/g DW [12], and 0.8 mg/g DW [12] to 16.8 mg/g DW [15], for LDG and GGCG, respectively, depending on culture conditions.

The highest level of SDG (5.55 ± 0.23 mg/g DW) and LDG (11.92 ± 1.03 mg/g DW) was observed at day 40 of adventitious root culture (Figure 7b). On the other hand, callus culture showed its highest accumulation level of SDG (2.23 ± 0.46 mg/g DW) and LDG (7.51 ± 1.06 mg/g DW) after 30 days of culture (Figure 7a). Both cultures showed the highest accumulation of lignans in their exponential growth phases, which is in harmony with the results of Wang et al. [5] in maturing seeds of *L. usitatissimum*. The dynamics of lignans accumulation showed that the adventitious root culture was more efficient for lignans production than the callus culture of *L. usitatissimum*. SDG accumulation was 2-fold higher, while LDG was 1.5-fold higher in adventitious root culture than callus culture. These findings are in coherence with those described by Gabr et al. [31] The authors reported that the accumulation of lignans was greater in hairy root cultures than the callus cultures of *L. usitatissimum*. Many reports are available in the literature, in which the adventitious root culture of many plants was more proficient in synthesizing bioactive compounds as compared to non-differentiated cell [39,41,47]. Contrary to our results, low levels of lignans were detected in the fungal elicited cell culture of *L. usitatissimum*, which might be due to the fungal-induced suppression of certain genes involved in the biosynthesis pathway of lignans [22,62]. In earlier reports, the cell cultures of flax have been established for the production of lignans, but these cultures accumulated comparatively lower levels of lignans than neolignans [10,11,13,14,15,16]. In the current report, adventitious root cultures accumulated higher levels of lignans in comparison to the previously established cell cultures of *L. usitatissimum*, presenting the best alternative to cell culture for the in vitro production of high levels of lignans.

Similar to lignans dynamics, neolignans (DCG and GGCG) accumulation was also high in adventitious root as compared to callus culture. The highest concentrations of DCG (21.62 ± 1.80 mg/g DW) and GGCG (4.92 ± 0.56 mg/g DW) were accumulated at the 30th day of root culture (Figure 8b), whereas callus culture showed the highest levels of DCG (12.24 ± 1.09 mg/g DW) and GGCG (2.21 ± 0.08 mg/g DW) on day 15 of culture (Figure 8a). In agreement with our results, the highest accumulations of DCG and GGCG were also reported in the coniferin-fed cell culture of *L. usitatissimum* [10]. Similarly, in two other reports, the authors claimed the highest accumulation of DCG contents at day 15 in cell cultures of *L. usitatissimum* [22,60]. DCG and GGCG were not detected in wild seeds, while high levels of these neolignans were detected in the transgenic seed lines of *L. usitatissimum* [14,24]. Production kinetics of neolignans in cell cultures of flax elicited with yeast extract, chemogenic silver nanoparticles and biogenic zinc oxide nanoparticles have been reported, which further endorses our results [13,14,15,16]. In particular, in the present study, it was noticed that, after reaching their maximum levels, the accumulation of neolignans started decreasing after the 30th (adventitious root) and 15th day (callus) of cultures, whereas the accumulation of lignans start increasing and reached their maximum values at days 40 and 30 of adventitious root and callus culture, respectively. This might be due to a controlled partition regulation of E-coniferyl alcohol toward the production of lignin, lignans and neolignans that share this common precursor for their biosynthesis [10,22]. The production of neolignans was linked with the activity of pinoresinol–lariciresinol reductases (PLR) and phenylcoumaran benzylic ether reductase (PCBER) enzymes, which have been isolated and characterized in several in vitro cultures of flax and play roles in lignans and neolignans biosynthesis [22,23,60].

## 3. Materials and Methods

### 3.1. Chemicals and Reagents

Exempt when mentioned, all chemicals and reagents were from Sigma-Aldrich (Sigma-Aldrich, Saint-Quentin Fallavier, France).

### 3.2. Plant Material and Establishment of Callus Culture

The *Linum usitatissimum* L. seeds (brown variety) were surface sterilized and inoculated on Murashige and Skoog basal media (MS0) [68], as reported by Anjum and Abbasi (2016) [55]. In order to generate callus cultures, root explants of approximately 1.0 cm size were cultured on MS media supplied with 2, 4-dichlorophenoxyacetic acid (2, 4-D), indole butyric acid (IBA), indole acetic acid (IAA), and α-naphthalene acetic acid (NAA) in variable concentrations (0.5–3.0 mg/L). MS0 media devoid of any phytohormones served as a control. These cultures were grown in a growth room under controlled temperature (25 ± 2 °C) and photoperiod (16/8 h light/dark). The experiment was carried out in triplicate culture flasks for each PGRs’ concentration and the whole experiment was conducted twice. Data regarding callus induction frequencies were noted on a daily basis and were subcultured after 21 days of culture using similar PGRs supplements. After five weeks of sub-culturing, respective calli were collected and subjected to fresh weight (FW) and dry weight (DW) determination.

### 3.3. Optimization and Growth Dynamics of Callus Culture

Data regarding different parameters of calli (Table 1) showed that the callus treated with NAA (1.0 mg L^−1^) exhibited the maximum level of callogenesis frequency, fresh biomass and dry biomass accumulation after five weeks of subculture. On this basis, a NAA (1.0 mg/L)-induced callus was selected to carry out its growth dynamics, in order to investigate the growth phase dependent accumulation of associated secondary plant metabolites in the callus culture of *L. usitatissimum*. To investigate growth dynamic, 1 g of a five-weeks-old callus was sub-cultured on fresh media using a similar concentration of NAA, under controlled conditions in a growth chamber. Data related to the growth dynamics were collected for a 45 days period with a five days’ interval.

### 3.4. Establishment of Adventitious Root Culture

For the initiation of adventitious root culture, approximately 2–3 cm root fragments were taken from three weeks old in vitro derived plantlets of *L. usitatissimum*. The root fragments were cultured into MS liquid media, augmented with a range of concentrations of auxin (0.5–3.0 mg/L of IBA, NAA and IAA). Each culture was established in 100 mL flasks containing approximately 35–40 mL of culture medium, and placed on a gyratory shaker at 120 rpm, under dark conditions. No auxin was added to MS0 media in control culture. The frequencies of adventitious root induction were recorded on a daily basis and the roots developed from the explants were sub-cultured after four weeks on MS medium, with the same auxins supplements, under the same culture conditions. To determine the role of different types of auxin on the development of the root, the adventitious roots produced in response to different auxin treatments were collected after five weeks of subculture and weighed for biomass determination (FW and DW).

### 3.5. Optimization and Growth Dynamics of Adventitious Root Culture

Data regarding different parameters of adventitious root (Table 2) showed that the roots responded well to 0.5 mg/L of NAA, resulting in the highest percentage of root formation, fresh biomass and dry biomass accumulation, after five weeks of sub-culture. Based on these factors, 0.5 mg/L NAA-induced roots were chosen to carry out their growth dynamics, in order to investigate the growth phase dependent accumulation of plant secondary metabolites in the root culture of *L. usitatissimum*. To investigate growth dynamics, 0.5 g of five-weeks-old roots were sub-cultured on a fresh medium, using a similar concentration of NAA, and placed under the same conditions as described in the establishment of adventitious root culture. All growth dynamic parameters were recorded for 55 days, with five days’ interval.

### 3.6. Determination of Total Polyphenols Contents and Antioxidant Activity

With an interval of five days, the fresh calli and roots were harvested from their culture flasks for the determination of biomass accumulation. For FW determination, excess water was removed by pressing calli and roots between the layers of filter paper. Afterwards, the DW of roots and calli was determined by desiccating them in an oven at 45 °C for 24 h.

Polyphenolics contents [total phenolic content (TPC) and total flavonoid content (TFC)] and antioxidant activity of calli and roots were evaluated by subjecting each sample to extraction by following the procedure reported by Rukh et al. [69] with slight modifications. In brief, 200 mg sample of each dried callus was taken and mixed with 10 mL methanol (99.9%). This mixture was kept on rotary shaker at 50 rpm for 24 hrs, sonicated for 15 min and vortexed for 10 min, repeating this step twice after 30 min interval. Lastly, the mixture was centrifuged for 10 min at 7000 rpm while the resulting supernatant was syringe filtered (0.45 µm) and stored at 4 °C to use in future analysis. In order to determine antioxidant activity, 2,2-Diphenyl-1-picrylhydrazyl (DPPH) was used as reported by Anjum et al. [12].

TPC was determined spectrophotometrically by using the Foli–Ciocalteu reagent (FCR) reported by Fazal et al. [43]. Firstly, the calli/root extracts (0.03 mL) were mixed with FCR (0.03 mL; 2N) and sterile distilled water (2.55 mL). Before syringe filtration (0.45 µm), the reaction mixture was centrifuged for 15 min at 12,000 rpm. TPC were measured through a UV-Visible spectrophotometer (Shimadzu, Tokyo, Japan) at 630 nm. Known amounts of gallic acid (0–40 μg/mL; standard) were used for making the calibration curve and finding out the regression curve factor (*R*^2^ = 0.998). All the experiments were carried out in triplicate. Lastly, the following formula was used to determine total phenolic production (TPP);

Total phenolic production (mg/L) = DW (g/L) × TPC (mg/g)(1)

The protocol reported by Anjum et al. [12] was used to determine the flavonoid contents in terms of TFC by using a colorimetric assay. Firstly, the calli/root extracts (0.25 mL) were mixed with double purified water (1.25 mL) and 5% aluminum chloride (0.063 mL). After this, the mixture was combined with 1M aqueous solution of sodium hydroxide (0.5 mL) and placed in the dark for 30 min. Absorbance of the reaction mixture was checked by spectrophotometer at 510 nm for TFC determination. Different known amounts of quercetin (0–40 μg/mL; standard) were used to draw the calibration curve and to find out the regression factor (*R*^2^ = 0.998). All determinations were carried out in triplicate, and the total flavonoid production (TFP) was calculated by using the following formula;

Total flavonoid production (mg/L) = DW (g/L) × TFC (mg/g)(2)

### 3.7. HPLC (High Performance Liquid Chromatography) Analysis of Lignans and Neolignans

From the dried biomass, lignans and neolignans were separated by exploiting the methods of Corbin et al. [8] and Renouard et al. [70], respectively. Then, 500 mg of lyophilized materials were immersed in 20 mL of 80% methanol and sonicated (1 h) at 25 °C. After centrifugation, the supernatant was taken out and subsequently evaporated to dryness at 40 °C. To release aglycone residues, citrate-phosphate buffer (1 mL, pH 4.8) made up of β-glucosidase (5 unit/mL) was used. Before injecting, the prepared extract was passed through filter paper (0.45 μm), after being homogenized. Then, RP-HPLC was used, in which a Varian liquid chromatographic system (Agilent Technology, Les Ullis, France) was employed, composed of a Varian Prostar 230 pump, Degasser (Metachem Degasit), Varian Prostar 335 PAD, and a Varian Prostar 410 auto sampler, and driven with Galaxie software (v1.9.3.2). An RP-18 column (Purospher (Merck, Saint Quentin Fallavier, France); 5 μm; 250 × 4 mm) was used at 35 °C for separation procedures, according to the previous method [11]. Additionally, the same extract was used for the relative conformation of aglycones vs glycosides, without enzymatic treatment. Calibration curves were used for quantifying lignans and neolignans and the results were represented in mg of glucoside (lariciresinol diglucoside, secoisolariciresinol diglucoside, guaiacylglycerol-*β*-coniferyl alcohol and dehydrodiconiferyl alcohol glucoside), which was equal to per gram of the DW. Calibration curves, limits of detection and quantification, as well as the validation of the method, were described by Anjum et al. [11]

### 3.8. Statistical Analysis of Data

All of the experimental work was conducted in synchronization and the analysis of experimental data was executed by using an analysis of variance (ANOVA). The significance at *p* < 0.05 and SE (±) were calculated by using Statistix 8.1 (Statistix, Tallahassee, FL, USA). Furthermore, Origin 8.5 software (OriginLab, Northampton, MA, USA) was used to generate graphics with their mean data values and standard errors.

## 4. Conclusions

In our research, we have successfully established the growth dynamics of the callus and adventitious root culture of flax, for the optimum synthesis of polyphenolics, including nutritionally and commercially important lignans (SDG and LDG) and neolignans (DCG and GGCG). Adventitious root culture demonstrated its efficiency in the synthesis of SDG, LDG, DCG and GGCG. On the contrary, the callus culture showed higher levels of TFP than adventitious root culture. The identification of these flavonoids, as well as phenolic acids, deserves further investigations. As there is limited information regarding biosynthesis, accumulation, stereochemistry and regulation of lignans and neolignans in in vitro cultures of plants, this study also showed that adventitious root culture may be useful to fill this gap. Current findings also suggested that the adventitious root culture could be scaled-up into a bioreactor and elicited to enhance the production of lignans and neolignans on a commercial level, and can help to meet the dietary and pharmacological requirements for their production. Indeed, unlike hairy root cultures, adventitious root cultures are genetically stable and do not produce any toxic chemicals such as opines.

## Figures and Tables

**Table 1:** Effect of different concentrations of PGRs on growth parameters of callus culture of *Linum usitatissimum*, after five weeks of culture.

	Conc. (mg/L)	Days Required	Percentage Callogenesis (%)	Fresh Weight (g/L)	Dry Weight (g/L)
IAA	0.5	9 ± 0.13	55.28 ± 1.33	103.01 ± 4.14	4.77 ± 0.034
	1.0	10 ± 0.11	42.03 ± 1.62	96.21 ± 2.92	4.41 ± 0.061
	2.0	10 ± 0.21	-	-	-
	3.0	-	-	-	-
NAA	0.5	8 ± 0.16	95.21 ± 3.09	323.11 ± 7.43	12.65 ± 0.24
	**1.0**	**7 ± 0.19**	**98.07 ± 2.24**	**392.76 ± 8.27**	**13.26 ± 0.44**
	2.0	7 ± 0.14	82.83 ± 3.15	277.34 ± 6. 29	11.15 ± 0.63
	3.0	9 ± 0.20	76.34 ± 2.03	228.19 ± 4.95	10.03 ± 1.07
2,4-D	0.5	-	-	-	-
	1.0	10 ± 0.43	65.21 ± 2.09	146.01 ± 3.65	5.23 ± 0.32
	2.0	8 ± 0.61	74.09 ± 2.05	189.09 ± 3.22	6.01 ± 0.22
	3.0	7 ± 0.10	88.31 ± 2.85	206.04 ± 2.97	9.21 ± 0.50
IBA	0.5	9 ± 0.25	42.53 ± 2.32	79.21 ± 4.61	3.81 ± 0.02
	1.0	10 ± 0.12	38.47 ± 1.20	62.87 ± 4.22	3.02 ± 0.04
	2.0	-	-	-	-
	3.0	-	-	-	-
Control	MS0	-	-	-	-

“-” indicates no active callogenesis. Highest values were given in Table 1 in bold.

**Table 2:** Effect of different concentrations of auxins on adventitious roots growth parameters of *Linum usitatissimum* after five weeks of culture.

	Conc. (mg/L)	Days Required	Percentage Rooting (%)	Fresh Weight (g/L)	Dry Weight (g/L)
IAA	0.5	11 ± 0.24	65.36 ± 2.32	61.23 ± 2.44	3.67 ± 0.08
	1.0	12 ± 0.21	62.28 ± 1.48	56.12 ± 3.79	3.21 ± 0.03
	2.0	-	-	-	-
	3.0	-	-	-	-
NAA	**0.5**	**8 ± 0.13**	**100.00 ± 2.99**	**123.21 ± 4.57**	**10.52 ± 0.16**
	1.0	10 ± 0.36	86.52 ± 3.26	92.76 ± 2.38	7.26 ± 0.16
	2.0	10 ± 0.14	80.32 ± 2.65	77.34 ± 3. 34	4.15 ± 0.08
	3.0	-	-	-	-
IBA	0.5	9 ± 0.45	78.52 ± 4.57	83.21 ± 3.65	4.81 ± 0.10
	1.0	10 ± 0.22	72.59 ± 4.92	79.87 ± 5.32	4.62 ± 0.08
	2.0	11± 0.38	67.62 ± 3.25	61.25 ± 4.45	3.71 ± 0.06
	3.0	-	-	-	-
Control	MS0	-	-	-	-

“-” indicates no active adventitious root formation. Highest values were given in Table 2 in bold.

## References

[1] Lainé E., Hano C., Lamblin F. (2007). Les lignanes phytoestrogènes du lin sont-ils des bienfaiteurs méconnus?. Phytotherapie.

[2] Lamblin F., Hano C., Fliniaux O., Mesnard F., Fliniaux M.-A., Lainé É. (2008). Interest of lignans in prevention and treatment of cancers. Med. Sci..

[3] Lainé E., Hano C., Lamblin F., Knasmüller S., DeMarini D.M., Johnson I.T.C.G. (2009). Lignans. Chemoprevention of Cancer and DNA Damage by Dietary Factors.

[4] Szewczyk M., Abarzua S., Schlichting A., Nebe B., Piechulla B., Briese V., Richter D.-U. (2014). Effects of extracts from Linum usitatissimum on cell vitality, proliferation and cytotoxicity in human breast cancer cell lines. J. Med. Plants Res..

[5] Wang H., Wang J., Guo X., Brennan C.S., Li T., Fu X., Chen G., Liu R.H. (2016). Effect of germination on lignan biosynthesis, and antioxidant and antiproliferative activities in flaxseed (*Linum usitatissimum* L.). Food Chem..

[6] Bussmann R.W., Batsatsashvili K., Kikvidze Z., Paniagua-Zambrana N.Y., Khutsishvili M., Maisaia I., Sikharulidze S., Tchelidze D. (2019). *Linum usitatissimum* L. Linaceae. Ethnobot. Mt. Reg. Far East. Eur. Ural. North. Cauc. Turk. Iran..

[7] Oomah B.D. (2001). Flaxseed as a functional food source. J. Sci. Food Agric..

[8] Corbin C., Fidel T., Leclerc E.A., Barakzoy E., Sagot N., Falguiéres A., Renouard S., Blondeau J., Ferroud C., Doussot J. (2015). Development and validation of an efficient ultrasound assisted extraction of phenolic compounds from flax (*Linum usitatissimum* L.) seeds. Ultrason. Sonochem..

[9] Ramsay A., Fliniaux O., Quéro A., Molinié R., Demailly H., Hano C., Paetz C., Roscher A., Grand E., Kovensky J. (2017). Kinetics of the incorporation of the main phenolic compounds into the lignan macromolecule during flaxseed development. Food Chem..

[10] Beejmohun V., Fliniaux O., Grand É., Lamblin F., Bensaddek L., Christen P., Kovensky J., Fliniaux M.-A., Mesnard F. (2007). Microwave-assisted extraction of the main phenolic compounds in flaxseed. Phytochem. Anal..

[11] Anjum S., Abbasi B.H., Doussot J., Favre-réguillon A., Hano C., Haider B., Doussot J., Favre-réguillon A., Hano C. (2017). Effects of photoperiod regimes and ultraviolet-C radiations on biosynthesis of industrially important lignans and neolignans in cell cultures of Linum usitatissimum L. (Flax ). J. Photochem. Photobiol. B Biol..

[12] Anjum S., Abbasi B.H., Hano C. (2017). Trends in accumulation of pharmacologically important antioxidant-secondary metabolites in callus cultures of *Linum usitatissimum* L.. Plant. Cell. Tissue Organ. Cult..

[13] Zahir A., Ahmad W., Nadeem M., Giglioli-Guivarc’h N., Hano C., Abbasi B.H. (2018). In vitro cultures of *Linum usitatissimum* L.: Synergistic effects of mineral nutrients and photoperiod regimes on growth and biosynthesis of lignans and neolignans. J. Photochem. Photobiol. B Biol..

[14] Ahmad W., Zahir A., Nadeem M., Garros L., Drouet S., Renouard S., Doussot J., Giglioli-Guivarc’h N., Hano C., Abbasi B.H. (2019). Enhanced production of lignans and neolignans in chitosan-treated flax (*Linum usitatissimum* L.) cell cultures. Process. Biochem..

[15] Nadeem M., Ahmad W., Zahir A., Hano C., Abbasi B.H. (2019). Salicylic acid-enhanced biosynthesis of pharmacologically important lignans and neo lignans in cell suspension culture of *Linum ussitatsimum* L.. Eng. Life Sci..

[16] Zahir A., Nadeem M., Ahmad W., Giglioli-Guivarc’h N., Hano C., Abbasi B.H. (2019). Chemogenic silver nanoparticles enhance lignans and neolignans in cell suspension cultures of *Linum usitatissimum* L.. Plant. Cell Tissue Organ. Cult..

[17] Ford J.D., Huang K.S., Wang H.B., Davin L.B., Lewis N.G. (2001). Biosynthetic pathway to the cancer chemopreventive secoisolariciresinol diglucoside-hydroxymethyl glutaryl ester-linked lignan oligomers in flax (*Linum usitatissimum*) seed. J. Nat. Prod..

[18] Dalisay D.S., Kim K.W., Lee C., Yang H., Rübel O., Bowen B.P., Davin L.B., Lewis N.G. (2015). Dirigent Protein-Mediated Lignan and Cyanogenic Glucoside Formation in Flax Seed: Integrated Omics and MALDI Mass Spectrometry Imaging. J. Nat. Prod..

[19] Corbin C., Drouet S., Markulin L., Auguin D., Lainé É., Davin L.B.L.B., Cort J.R.J.R., Lewis N.G.N.G., Hano C. (2018). A genome-wide analysis of the flax (*Linum usitatissimum* L.) dirigent protein family: From gene identification and evolution to differential regulation. Plant. Mol. Biol..

[20] Moinuddin S.G.A., Cort J.R., Smith C.A., Hano C., Davin L.B., Lewis N.G. (2019). Linum Lignan and Associated Biochemical Pathways in Human Health and Plant Defense. Plant Genetics and Genomics: Crops and Models.

[21] Von Heimendahl C.B.I., Schäfer K.M., Eklund P., Sjöholm R., Schmidt T.J., Fuss E. (2005). Pinoresinol–lariciresinol reductases with different stereospecificity from Linum album and Linum usitatissimum. Phytochemistry.

[22] Hano C., Addi M., Bensaddek L., Crônier D., Baltora-Rosset S., Doussot J., Maury S., Mesnard F., Chabbert B., Hawkins S. (2006). Differential accumulation of monolignol-derived compounds in elicited flax (*Linum usitatissimum*) cell suspension cultures. Planta.

[23] Hano C., Martin I., Fliniaux O., Legrand B., Gutierrez L., Arroo R.R.J., Mesnard F., Lamblin F., Lainé E. (2006). Pinoresinol-lariciresinol reductase gene expression and secoisolariciresinol diglucoside accumulation in developing flax (*Linum usitatissimum*) seeds. Planta.

[24] Renouard S., Tribalatc M., Lamblin F., Mongelard G., Fliniaux O., Corbin C., Marosevic D., Pilard S., Demailly H., Gutierrez L. (2014). RNAi-mediated pinoresinol lariciresinol reductase gene silencing in flax (*Linum usitatissimum* L.) seed coat: Consequences on lignans and neolignans accumulation. J. Plant. Physiol..

[25] Corbin C., Drouet S., Mateljak I., Markulin L., Decourtil C., Renouard S., Lopez T., Doussot J., Lamblin F., Auguin D. (2017). Functional characterization of the pinoresinol–lariciresinol reductase-2 gene reveals its roles in yatein biosynthesis and flax defense response. Planta.

[26] Hemmati S., Von Heimendahl C.B.I., Klaes M., Alfermann A.W., Schmidt T.J., Fuss E. (2010). Pinoresinol-Lariciresinol Reductases with Opposite Enantiospecificity Determine the Enantiomeric Composition of Lignans in the Different Organs of *Linum usitatissimum* L.. Planta Med..

[27] Markulin L., Corbin C., Renouard S., Drouet S., Gutierrez L., Mateljak I., Auguin D., Hano C., Fuss E., Lainé E. (2019). Pinoresinol–lariciresinol reductases, key to the lignan synthesis in plants. Planta.

[28] Ghose K., Selvaraj K., McCallum J., Kirby C.W., Sweeney-Nixon M., Cloutier S.J., Deyholos M., Datla R., Fofana B. (2014). Identification and functional characterization of a flax UDP-glycosyltransferase glucosylating secoisolariciresinol (SECO) into secoisolariciresinol monoglucoside (SMG) and diglucoside (SDG). BMC Plant. Biol..

[29] Teponno R.B., Kusari S., Spiteller M. (2016). Recent advances in research on lignans and neolignans. Nat. Prod. Rep..

[30] Fofana B., Ghose K., McCallum J., You F.M., Cloutier S. (2017). UGT74S1 is the key player in controlling secoisolariciresinol diglucoside (SDG) formation in flax. BMC Plant Biol..

[31] Gabr A.M.M., Mabrok H.B., Ghanem K.Z., Blaut M., Smetanska I. (2016). Lignan accumulation in callus and Agrobacterium rhizogenes-mediated hairy root cultures of flax (Linum usitatissimum). Plant. Cell Tissue Organ. Cult..

[32] Gabr A.M.M., Mabrok H.B., Abdel-Rahim E.A., El-Bahr M.K., Smetanska I. (2018). Determination of lignans, phenolic acids and antioxidant capacity in transformed hairy root culture of Linum usitatissimum. Nat. Prod. Res..

[33] Markulin L., Corbin C., Renouard S., Drouet S., Durpoix C., Mathieu C., Lopez T., Auguin D., Hano C., Lainé É. (2019). Characterization of LuWRKY36, a flax transcription factor promoting secoisolariciresinol biosynthesis in response to Fusarium oxysporum elicitors in Linum usitatissimum L. hairy roots. Planta.

[34] Sertse D., You F.M., Ravichandran S., Cloutier S. (2019). The Complex Genetic Architecture of Early Root and Shoot Traits in Flax Revealed by Genome-Wide Association Analyses. Front. Plant. Sci..

[35] Siegień I., Adamczuk A., Wróblewska K. (2013). Light affects in vitro organogenesis of Linum usitatissimum L. and its cyanogenic potential. Acta Physiol. Plant..

[36] Millam S., Obert B., Pret’ová A. (2005). Plant cell and biotechnology studies in Linum usitatissimum—A review. Plant. Cell. Tissue Organ. Cult..

[37] Reis R.V., Borges A.P.P.L., Chierrito T.P.C., de Souto E.R., de Souza L.M., Iacomini M., de Oliveira A.J.B., Gonçalves R.A.C. (2011). Establishment of adventitious root culture of Stevia rebaudiana Bertoni in a roller bottle system. Plant. Cell Tissue Organ. Cult..

[38] Murthy H.N., Hahn E.J., Paek K.Y. (2008). Adventitious roots and secondary metabolism. Chin. J. Biotechnol..

[39] Khan M.A., Abbasi B.H., Shah N.A., Yücesan B., Ali H. (2015). Analysis of metabolic variations throughout growth and development of adventitious roots in Silybum marianum L.(Milk thistle), a medicinal plant. Plant. Cell Tissue Organ. Cult..

[40] Cui X.-H., Chakrabarty D., Lee E.-J., Paek K.-Y. (2010). Production of adventitious roots and secondary metabolites by Hypericum perforatum L. in a bioreactor. Bioresour. Technol..

[41] Jeong J.-A., Wu C.-H., Murthy H.N., Hahn E.-J., Paek K.-Y. (2009). Application of an airlift bioreactor system for the production of adventitious root biomass and caffeic acid derivatives of Echinacea purpurea. Biotechnol. Bioprocess. Eng..

[42] Praveen N., Murthy H.N. (2010). Production of withanolide-A from adventitious root cultures of Withania somnifera. Acta Physiol. Plant..

[43] Fazal H., Abbasi B.H., Ahmad N. (2014). Optimization of adventitious root culture for production of biomass and secondary metabolites in *Prunella vulgaris* L.. Appl. Biochem. Biotechnol..

[44] Janowicz J., Niemann J., Wojciechowski A. (2012). The effect of growth regulators on the regeneration ability of flax (*Linum usitatissimum* L.) hypocotyl explants in in vitro culture. Biotechnol. J. Biotechnol. Comput. Biol. Bionanotechnol..

[45] Rasool R., Ganai B.A., Kamili A.N., Akbar S. (2012). Antioxidant potential in callus culture of Artemisia amygdalina Decne. Nat. Prod. Res..

[46] Raj D., Kokotkiewicz A., Drys A., Luczkiewicz M. (2015). Effect of plant growth regulators on the accumulation of indolizidine alkaloids in Securinega suffruticosa callus cultures. Plant. Cell Tissue Organ. Cult..

[47] Kim Y.-S., Hahn E.-J., Murthy H.N., Paek K. (2004). Adventitious root growth and ginsenoside accumulation in Panax ginseng cultures as affected by methyl jasmonate. Biotechnol. Lett..

[48] Zhao J., Zhu W.-H., Hu Q., He X.-W. (2001). Enhanced indole alkaloid production in suspension compact callus clusters of Catharanthus roseus: Impacts of plant growth regulators and sucrose. Plant. Growth Regul..

[49] Abbasi B.H., Siddiquah A., Tungmunnithum D., Bose S., Younas M., Garros L., Drouet S., Giglioli-Guivarc’h N., Hano C. (2019). Isodon rugosus (Wall. ex Benth.) codd in vitro cultures: Establishment, phytochemical characterization and in vitro antioxidant and anti-aging activities. Int. J. Mol. Sci..

[50] Kikowska M., Thiem B., Sliwinska E., Rewers M., Kowalczyk M., Stochmal A., Oleszek W. (2014). The Effect of Nutritional Factors and Plant Growth Regulators on Micropropagation and Production of Phenolic Acids and Saponins from Plantlets and Adventitious Root Cultures of *Eryngium maritimum* L.. J. Plant. Growth Regul..

[51] Ali M., Abbasi B.H. (2013). Production of commercially important secondary metabolites and antioxidant activity in cell suspension cultures of *Artemisia absinthium* L.. Ind. Crop. Prod..

[52] Lindsey K., Yeoman M.M. (1983). The relationship between growth rate, differentiation and alkaloid accumulation in cell cultures. J. Exp. Bot..

[53] Rice-Evans C.A., Miller N.J. (1996). Antioxidant activities of flavonoids as bioactive components of food. Biochem. Soc. Trans..

[54] Misra N., Misra P., Datta S.K., Mehrotra S. (2005). In vitro biosynthesis of antioxidants from Hemidesmus indicus R. Br. cultures. Vitr. Cell. Dev. Biol..

[55] Anjum S., Abbasi B.H. (2016). Thidiazuron-enhanced biosynthesis and antimicrobial efficacy of silver nanoparticles via improving phytochemical reducing potential in callus culture of *Linum usitatissimum* L.. Int. J. Nanomed..

[56] Andreazza N.L., Abreu I.N., Sawaya A., Eberlin M.N., Mazzafera P. (2009). Production of imidazole alkaloids in cell cultures of jaborandi as affected by the medium pH. Biotechnol. Lett..

[57] Zahir A., Abbasi B.H., Adil M., Anjum S., Zia M. (2014). Synergistic effects of drought stress and photoperiods on phenology and secondary metabolism of *Silybum marianum*. Appl. Biochem. Biotechnol..

[58] Struijs K., Vincken J.-P., Doeswijk T.G., Voragen A.G.J., Gruppen H. (2009). The chain length of lignan macromolecule from flaxseed hulls is determined by the incorporation of coumaric acid glucosides and ferulic acid glucosides. Phytochemistry.

[59] Corbin C. (2015). Etude de la Régulation Transcriptionnelle de la Synthèse des Lignanes du lin (*Linum usitatissimum* L.). PhD Thesis.

[60] Attoumbré J., Charlet S., Baltora-Rosset S., Hano C., Raynaud-Le Grandic S., Gillet F., Bensaddek L., Mesnard F., Fliniaux M.A. (2006). High accumulation of dehydrodiconiferyl alcohol-4-β-D-glucoside in free and immobilized Linum usitatissimum cell cultures. Plant. Cell Rep..

[61] Attoumbre J., Hano C., Mesnard F., Lamblin F., Bensaddek L., Raynaud-Le Grandic S., Laine É., Fliniaux M.-A., Baltora-Rosset S. (2006). Identification by NMR and accumulation of a neolignan, the dehydrodiconiferyl alcohol-4-$β$-D-glucoside, in Linum usitatissimum cell cultures. Comptes. Rendus Chim..

[62] Hano C., Addi M., Fliniaux O., Bensaddek L., Duverger E., Mesnard F., Lamblin F., Lainé E. (2008). Molecular characterization of cell death induced by a compatible interaction between Fusarium oxysporum f. sp. linii and flax (*Linum usitatissimum*) cells. Plant. Physiol. Biochem..

[63] Corbin C., Renouard S., Lopez T., Lamblin F., Lainé E., Hano C. (2013). Identification and characterization of cis-acting elements involved in the regulation of ABA- and/or GA-mediated LuPLR1 gene expression and lignan biosynthesis in flax (*Linum usitatissimum* L.) cell cultures. J. Plant. Physiol..

[64] Corbin C., Decourtil C., Marosevic D., Bailly M., Lopez T., Renouard S., Doussot J., Dutilleul C., Auguin D., Giglioli-Guivarc’h N. (2013). Role of protein farnesylation events in the ABA-mediated regulation of the Pinoresinol-Lariciresinol Reductase 1 (LuPLR1) gene expression and lignan biosynthesis in flax (*Linum usitatissimum* L.). Plant. Physiol. Biochem..

[65] Markulin L., Drouet S., Corbin C., Decourtil C., Garros L., Renouard S., Lopez T., Mongelard G., Gutierrez L., Auguin D. (2019). The control exerted by ABA on lignan biosynthesis in flax (*Linum usitatissimum* L.) is modulated by a Ca 2+ signal transduction involving the calmodulin-like LuCML15b. J. Plant. Physiol..

[66] Beejmohun V., Fliniaux O., Hano C., Pilard S., Grand E., Lesur D., Cailleu D., Lamblin F., Lainé E., Kovensky J. (2007). Coniferin dimerisation in lignan biosynthesis in flax cells. Phytochemistry.

[67] Nadeem M., Abbasi B.H., Garros L., Drouet S., Zahir A., Ahmad W., Giglioli-Guivarc’h N., Hano C. (2018). Yeast-extract improved biosynthesis of lignans and neolignans in cell suspension cultures of *Linum usitatissimum* L.. Plant. Cell. Tissue Organ. Cult..

[68] Murashige T., Skoog F. (1962). A Revised Medium for Rapid Growth and Bio Assays with Tobacco Tissue Cultures. Physiol. Plant..

[69] Rukh G., Ahmad N., Rab A., Ahmad N., Fazal H., Akbar F., Ullah I., Mukhtar S., Samad N. (2019). Photo-dependent somatic embryogenesis from non-embryogenic calli and its polyphenolics content in high-valued medicinal plant of Ajuga bracteosa. J. Photochem. Photobiol. B Biol..

[70] Renouard S., Hano C., Corbin C., Fliniaux O., Lopez T., Montguillon J., Barakzoy E., Mesnard F., Lamblin F., Lainé E. (2010). Cellulase-assisted release of secoisolariciresinol from extracts of flax (*Linum usitatissimum*) hulls and whole seeds. Food Chem..

